# Treatment options for recurrent platinum-resistant ovarian cancer: A systematic review and Bayesian network meta-analysis based on RCTs

**DOI:** 10.3389/fonc.2023.1114484

**Published:** 2023-04-11

**Authors:** Juan Li, Guorong Zou, Wei Wang, Chen Yin, Haowen Yan, Shengpeng Liu

**Affiliations:** ^1^ Department of Oncology, Guangzhou Panyu District Central Hospital, Guangzhou, China; ^2^ Department of Nursing, Central Hospital of Gansu Province, Lanzhou, China; ^3^ Department of Clinical Medicine, People’s Hospital of Weining County, Bijie, China

**Keywords:** ovarian neoplasms recurrence, randomized controlled trial, blind RCT, systematic review, Bayesian network meta-analysis

## Abstract

**Background:**

There are a variety of treatment options for recurrent platinum-resistant ovarian cancer, and the optimal specific treatment still remains to be determined. Therefore, this Bayesian network meta-analysis was conducted to investigate the optimal treatment options for recurrent platinum-resistant ovarian cancer.

**Methods:**

Pubmed, Cochrane, Embase, and Web of Science were searched for articles published until 15 June 2022. The outcome measures for this meta-analysis were overall survival (OS), progression-free survival (PFS), and adverse events (AEs) of Grade 3-4. The Cochrane assessment tool for risk of bias was used to evaluate the risk of bias of the included original studies. The Bayesian network meta-analysis was conducted. This study was registered on PROSPERO (CRD42022347273).

**Results:**

Our systematic review included 11 RCTs involving 1871 patients and 11 treatments other than chemotherapy. The results of meta-analysis showed that the overall survival (OS) was the highest in adavosertib + gemcitabine compared with conventional chemotherapy, (HR=0.56,95%CI:0.35-0.91), followed by sorafenib + topotecan (HR=0.65, 95%CI:0.45-0.93). In addition, Adavosertib + Gemcitabine regimen had the highest PFS (HR=0.55,95%CI:0.34-0.88), followed by Bevacizumab + Gemcitabine regimen (HR=0.48,95%CI:0.38-0.60) and the immunotherapy of nivolumab was the safest (HR=0.164,95%CI:0.312-0.871) with least adverse events of Grades 3-4.

**Conclusions:**

The results of this study indicated that Adavosertib (WEE1 kinase-inhibitor) + gemcitabine regimen and Bevacizumab + Gemcitabine regimen would be significantly beneficial to patients with recurrent platinum-resistant ovarian cancer, and could be preferred for recurrent platinum-resistant ovarian cancer. The immunotherapeutic agent, Nivolumab, is of considerable safety, with a low risk for grade-III or IV adverse events. Its safety is comparable to Adavosertib + gemcitabine regimen. Pazopanib + Paclitaxel (weekly regimen), Sorafenib + Topotecan/Nivolumab could be selected if there are contraindications of the above strategies.

**Systematic review registration:**

https://www.crd.york.ac.uk/prospero/, identifier CRD42022347273.

## Introduction

Ovarian cancer (OC) still remains to be a fatal gynecological cancer. As a common aggressive cancer in female genital tract, OC is second only to uterine cancer in the United States, with an estimated 21,880 cases diagnosed every year. Approximately 13,850 women died of ovarian cancer annually, which is the most common cause of death for women with gynecological malignancies (1). Although significant progress has been made in OC treatment, relapse occurs in approximately 85% of ovarian cancer patients who eventually develop resistance to chemotherapy (2). Therefore, platinum-free interval (PFI) is an important predictor for success in treating recurrent ovarian cancer (3). The fifth Gynecologic Cancer Intergroup (GCIG) recommends re-categorization of platinum sensitivity based on PFI duration (< 1 month, 1–6 months, 6–12 months, and > 12 months). However, platinum-resistance is defined according to the interval between the most recent platinum therapy and the recurrence less than 6 months (4).

First-line treatment for advanced ovarian cancer includes tumorectomy and platinum-based chemotherapy with or without anti-angiogenic therapy or PARP inhibitors (5). Platinum resistance, whether generated initially or acquired, is a major obstacle in treating ovarian cancer. Novel therapeutic strategies are urgently needed to further improve clinical outcomes.

At present, the main therapeutic strategy for platinum-resistant patients with recurrent ovarian cancer is systemic chemotherapy, such as polyethylene glycol liposome doxorubicin (PLD) (6, 7) and topotecan (6). The effective rate in most patients is 10–30% (7). The effective rate of weekly paclitaxel chemotherapy is 25–55% (8). Other chemotherapy regimens include gemcitabine (9, 10), etoposide (11), ifosfamide, docetaxel, and vinorelbine, with an overall effective rate of 10% to 20%, a median progression-free survival (PFS) of 3 to 4 months, and a median overall survival (OS) of 9 to 12 months (12). Treatment option depends on previous treatment history, patients’ characteristics, and side effects of each drug. There is an urgent need for more treatment options.

Compared with using chemotherapy alone, anti-angiogenic agents, targeted therapy, and immunotherapy can provide more benefits in survival rate for recurrent platinum resistance. Although various anti-cancer agents are used in treating recurrent platinum-resistant ovarian cancer, there is no consensus on the international standards at present. Therefore, it is of necessity to simultaneously compare the efficacy and tolerance of multiple treatments, including chemotherapy, targeted therapy, anti-angiogenic agents and immunotherapy, to provide more available treatment options for recurrent platinum-resistant ovarian cancer. However, there is still a lack of analysis of treatment options for relapsed platinum-resistant ovarian cancer. A network meta-analysis is required to comprehensively compare multiple treatment options for recurrent platinum-resistant ovarian cancer.

Currently, there are two types of network meta-analysis, namely Frequentist-based network meta-analysis (13) and Bayesian-based network meta-analysis (14). The main difference between the two lies in their inconsistent cognition of estimated parameters. The Frequentists believe that estimated parameters are fixed, but observation experiments are limited. Hence, the 95% confidence interval, assuming that an infinite number of observation experiments are performed, is calculated. In contrast, the Bayesians believe that estimated parameters are random variables. Their inference depends completely on the posterior distribution of the random variables, and all statistical properties of random variables are determined by the posterior distribution. Usually, the two approaches are neither superior nor inferior to each other. Nonetheless, in the present network meta-analysis, the Bayesian method has some unique advantages, such as high flexibility and natural decision-making models. Besides, current network meta-analyses are mostly based on the Bayesian framework.

This study aimed to conduct a Bayesian network meta-analysis to evaluate and rank multiple treatment options for recurrent platinum-resistant ovarian cancer (PROC). The results could provide reference to clinical decision-making in choosing the optimal therapeutic strategies for the patients with recurrent platinum-resistant ovarian cancer in the future.

## Methods

This network meta-analysis was reported following the Network Meta-analysis Statement in Preferred Reporting Items for System Review and Meta-analysis (PRISMA2020). This study was prospectively registered on Prospero (CRD42022347273).

### Retrieval strategy

Pubmed, Cochrane, Embase, and Web of science were searched for randomized controlled trials (RCTs) on therapeutic strategies for recurrent platinum-resistant ovarian cancer published until June 15, 2022. The search method was subject terms + free words. There was no restriction on regions. Detailed search strategy was presented in **Appendix 1**.

### Inclusion and exclusion criteria

Articles were considered eligible based on the following criteria: 1) Patients with ovarian cancer had a recurrence within 6 months of postoperative platinum chemotherapy; 2) Outcome measures include one of the following items: overall survival (OS), progression-free survival (PFS), adverse events (AEs) of grade 3-4, and stage 2 or 3 RCTs.

Exclusion criteria: 1) Publications including non-randomized controlled trials, one-arm design studies, dose finding studies, conference abstracts, and systematic reviews or meta-analyses; 2) The full text of the research is unavailable; 3) If there are multiples original studies investigating the same outcome measure based on the same RCT, the study with larger sample size is included.

### Literature screening and data extraction

The retrieved articles were imported into Endnote. After removing the duplication, the preliminarily eligible studies were screened by reading the titles and abstracts, and the full text was downloaded. The eligible studies were finally determined by reading the full text. Before information extraction, we created a standard data extraction spreadsheet which included the information on the trials 1) the first author, publication year, trial name, design type, and author’s nationality); 2) The information on stage, intervention method, sample size of each intervention group, and total sample size; 3) Baseline characteristics of the patient, including median age and treatment history; 4) Primary or secondary endpoints, including OS, PFS, AE of Grade 3-4. The aforementioned literature screening and information extraction were conducted independently by two investigators (LJ, YHW), and cross-checked after completion. If there was any dissent, a third investigator (YC) was consulted for determination.

### Assessment of bias

Two investigators used the Cochrane Collaboration assessment tool for risk of bias of RCTs to assess the risk of bias. The assessment tool contained the following 7 items: random sequence generation, allocation concealment, blinding of subjects and intervention providers, blinding of result reviewers, incomplete data, selective reporting, and other sources of bias. Each item was rated as low bias, high bias, or unclear. The above literature screening and information extraction were conducted independently by two investigators (LJ, YHW), and cross-examined after completion. If there was any dissent, a third investigator (YC) was consulted for determination.

### Outcome indicators

The primary outcome measure was OS, and secondary outcome measures were PFS and AEs. The hazard ratio (HR) and 95% confidence interval (CI) of the treatment in each study were compared to calculate the effect size of the survival outcomes (e.g., OS and PFS), whereas risk ratio (RR) was applied for AEs. If HR and the 95% CI were not reported in a study, the GetData Graph Digitizer software (version 2.26; Http://getdata-graph-digitizer.com) was used according to the method described by Tierney et al. to extract the value from the reported Kaplan-Meier curve and the number of high-risk patients at each time interval (Accumulative survival) (15) AEs was defined as treatment-related adverse events of Grade 3-5 according to the National Cancer Institute Common Terminology Criteria for Adverse Events (CTCAE) version 3.0 or 4.03. If the TRAE was not reported, reports of Grade 3-4 AEs would be included for network meta-analysis. Progression-free survival (PFS) was defined as the time between randomization and disease-progression, death from any cause, or the last assessment without progression. Overall survival (OS) was defined as the time between randomization and death or the date of the lost follow-up.

### Statistical analysis

This network meta-analysis adopted Bayesian random-effects model to compare the effects of interventions to determine their effectiveness. The Markov chain Monte Carlo method was used for creating the model. Four Markov chains were run at the same time, and the annealing time was set as 20000 times. The modeling was completed after 50000 simulation iterations. The model fitting and the global consistency were compared using the deviance information criterion (DIC). If there was a closed-loop network, the node splitting method was used to analyze the local consistency. Additionally, interventions were ranked based on the surface under the cumulative ranking curve.

(SUCRA) and the league table was generated to compare differences in effects between different interventions. Funnel plots were used to visualize the heterogeneity between studies. Analyses were performed by using Stata 15.0(Stata Corporation, College Station, TX) and R 4.2.0 (R Development Core Team, Vienna, Http://www.R-project.org). P<0.05 indicated that the difference was statistically significant.

## Results

### Results of literature retrieval

**Figure 1:**
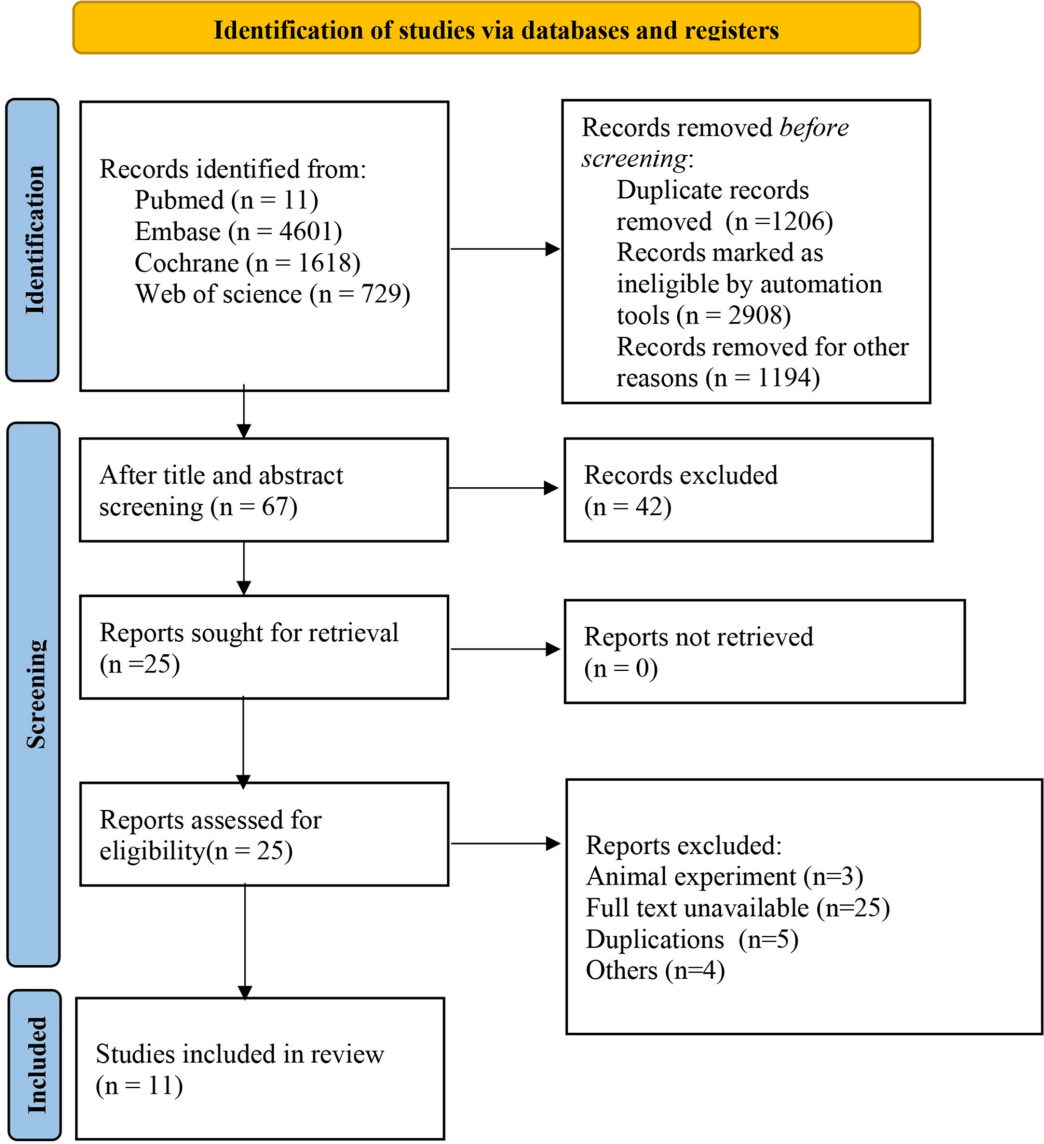
Literature screening flowchart. Other reasons: inconsistent intervention, inconsistent population, animal experiments, meta-analyses, conference abstracts, online publications, reviews, editorial materials, retrospective studies, non-RCTs, etc.

Initially, 11540 studies were retrieved through literature search, and duplications were removed. Afterwards, 10253 articles were screened out according to the title and abstract, and 42 were selected for full-text review. Among the remaining studies that might be eligible, 31 studies were excluded. Finally, 11 high-quality RCT with delicate design (complete studies published between 2013 and 2022, including 1687 patients with recurrent platinum-resistant ovarian cancer) were included in this network meta-analysis. A flowchart describing the literature selection process is shown in **Figure 1**.

### Basic characteristics of the included studies

**Figure 2:**
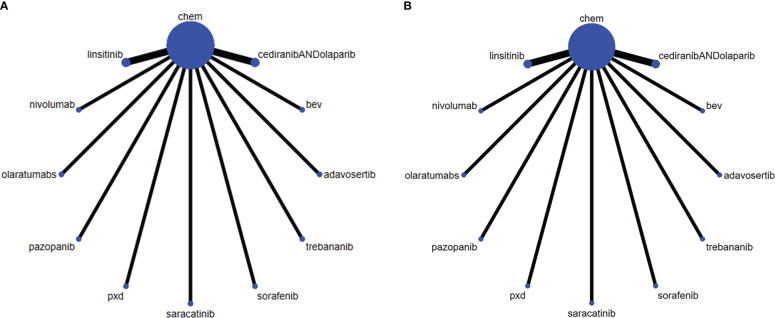
The network of each intervention for **(A)** PFS and **(B)** OS. The width of the lines represents the number of studies under direct comparison. The thicker the line, the greater the number of studies.

The randomized controlled trials included 4 Phase 3 trials (16–19), and 7 Phase 2 trials (20–26). Three treatment categories were reported, including chemotherapy (Paclitaxel, carbo, CT (Pegylated Liposomal doxorubicin, paclitaxel, topotecan, TC protocol (Topotecan, Pegylated Liposomal doxorubicin, paclitaxel, or gemcitabine), immunotherapy (olaratumabs, nivolumab), targeted therapy (intermittent linsitinib+chem vs consecutive linsitinib+chem), PDX (Phenonoxodiol+Carboplatin vs Carboplatin), and bev (CT+bevacizumab vs CT, Saracatinib+ paclitaxel, Pazopanib+ paclitaxel, Trebananib+ liposome doxorubicin, Sorafenib+ topotecan, Adavosertib+ gemcitabine, cediranib+olaparib vs paclitaxel). The network for analyzing all the treatment methods is shown in **Figure 2** (PFS, OS). The general characteristics of the included RCTs are presented in **Table 1** in detail.

**Table 1:** Basic characteristics of the literature.

First author	Year of publication	Study design	Country	Source of patients	Interventions	Number of cases	Age
Amit Oza (19)	2018	Phase 2 trial	Canada	Single-center	intermittent linsitinib+paclitaxel continuous linsitinib+paclitaxel paclitaxel	51 51 50	57.75
C. Fotopoulou (15)	2013	phase III trial	United Kingdom	Multi-center	phenoxodiol (PXD)-resistance reversal potential +AUC2-carboplatin Placebo+AUC2-carboplatin	70 72	57.5 (39–78) 59.0 (37–82)
Eric Pujade-Lauraine (16)	2014	Phase III Trial	America	Single-center	single-agent chemotherapy Single-agent chemotherapy+bevacizumab	182 179	61(25-84) 62 (25-80)
I. A. McNeish (27)	2014	phase II trial	United Kingdom	Single-center	Paclitaxel + saracatinib (wPxl+S) weekly Paclitaxel + placebo (wPxl+P)	71 36	63.4 (20.0–82.1)
Sandro Pignata (20)	2015	phase 2 trial	Italy	Multi-center	Paclitaxel Paclitaxel+pazopanib	37 37	58 (53–68) 56 (52–65)
Christian Marth (17)	2017	Phase III	Austria	Multi-center	Placebo+PLD Trebananib+liposome doxorubicin	114 109	61 (53 68) 60 (53 66)
Radoslav Chekerov, Felix Hilpert (21)	2018	phase 2 trial	Germany	Multi-center	Sorafenib+ topotecan Placebo+ topotecan	83 89	59 (31–78) 58 (25–79)
William P. McGuire (28)	2018	phase II study	America	Multi-center	olaratumab+liposome doxorubicin liposome doxorubicin	62 61	58.7 (10.07) 59.8 (9.70)
Stephanie Lheureux, Mihaela (22)	2021	phase 2 trial	Canada	Multi-center	Adavosertib+gemcitabine Placebo+gemcitabine	99 25	62 (54–67)
Junzo Hamanishi, MD (18)	2021	phase III trial	Japan	Multi-center	Nivolumab Chemotherapy	157 159	58.0 (29, 84) 60.0 (34, 80)
Nicoletta Colombo (23)	2022	phase II trial	Italy	Multi-center	Paclitaxel Cediranib+olaparib	41 41 41	62.5 (56.6–69.7) 64.2 (54.0–68.4)
Amit Oza (29)	2020	open-label phase 2	Canada	Multi-center	Gemcitabine+carboplatin TC TC crossover(TC to GC)	51 49	62

### Assessment of risk of bias

**Figure 3:**
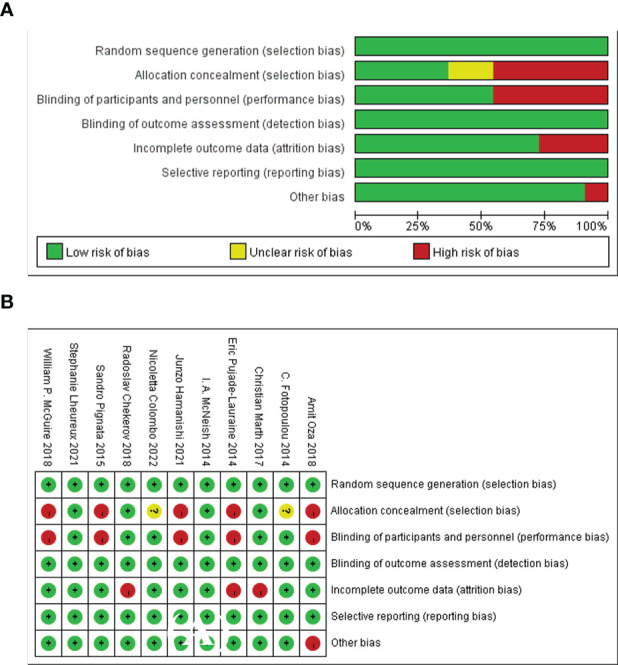
The assessment of risk of bias for the included studies. **(A)** Summary risk of bias, **(B)** Detailed risk of bias in each study.

The risk of bias of the included RCTs was generally rated as low risk for most items. Random sequence generation and allocation concealment in 11(85%) trials were rated as low-risk since the authors described the randomization method in detail. Two open-label RCTs (15%) with double-blinding of participants and subjects were considered as high-risk. Blinding evaluation of the results, incomplete data and selective reporting in all the included RCTs were rated as low-risk. However, a total of 2 RCTs (15%) were assessed as high-risk for other bias. In summary, Amit Oza (17) was considered to be the study with the highest risk of bias, because it had two items rated as unclear risks. Therefore, the study by Amit Oza was not used in the sensitivity analysis. This study summarized the assessment of the risk of bias and provided detailed evidence for determining the risk of each randomized controlled trial (**Figure 3**).

### The primary outcome measure: OS

**Figure 4:**
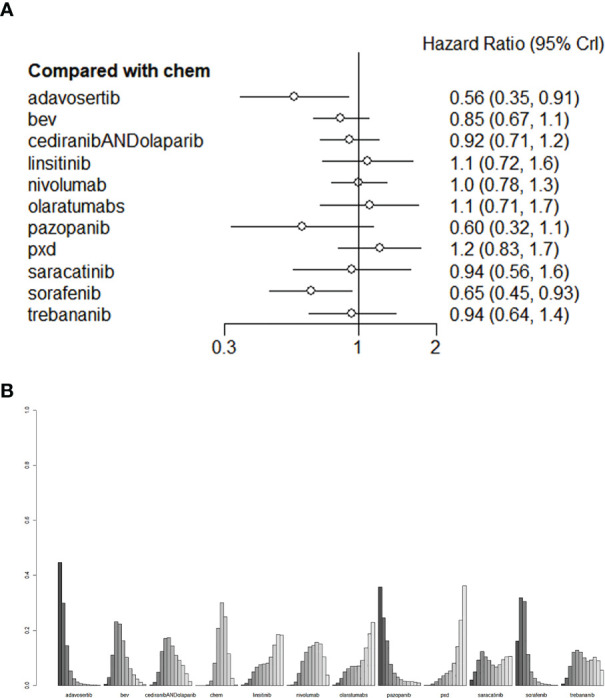
**(A)** The forest plot and **(B)** the ranking plot for OS. The color of the lines in **Figure 4B** from dark to light represents the ranking from the best to the worst. A darker color represents a higher probability of becoming the best intervention, and a lighter color represents a lower probability. The cediranibANDcediranib stands for combination medication.

In this network analysis, we comprehensively compared 10 individual treatment nodes, including 2 immunotherapy nodes (nivolumab 240 mg Qw2, olaratumabs 20 mg/kg, Qw2), and 9 agents for targeted therapies (olaparib, Trebananib 15mg/kg, Institute, phenoxodiol, Saracatinib, adavosertib, Cediranib, sorafenib 400mg, pazopanib 800mg qd). Conventional chemotherapy Adavosertib 175 mg + gemcitabine (HR=0.56,95%CI:0.35-0.91) ranked the first overall survival, followed by Sorafenib400 mg+ topotecan (HR=0.65,95%CI:0.45-0.93), with statistically significant difference, which suggested that this regimen could evidently improve the OS of recurrent PROC patients. (liposome doxorubicin or weekly paclitaxel or topotecan) had an HR of 0.85 (95%CI:0.67-1.1) and Cediranib+Olapalib had an HR of 0.92 (95% CI: 0.71-1.2). There was no significant difference in OS (**Figure 4A**). The league table depicting the relative effects of all treatment pairs on OS is shown in **Figure 4B**. We performed validation through frequency-based network meta-analysis, and the results showed that Adavosertib + Gemcitabine regimen (HR=0.56, 95%CI: 0.35-0.90) and sorafenib + topotecan regimen (HR=0.65, 95%CI: 0.45-0.93) significantly improved the OS. Detailed results of frequency-based network meta-analysis are provided in **Appendix 2**.

### The secondary outcome measures: Progression-free survival and adverse events

**Figure 5:**
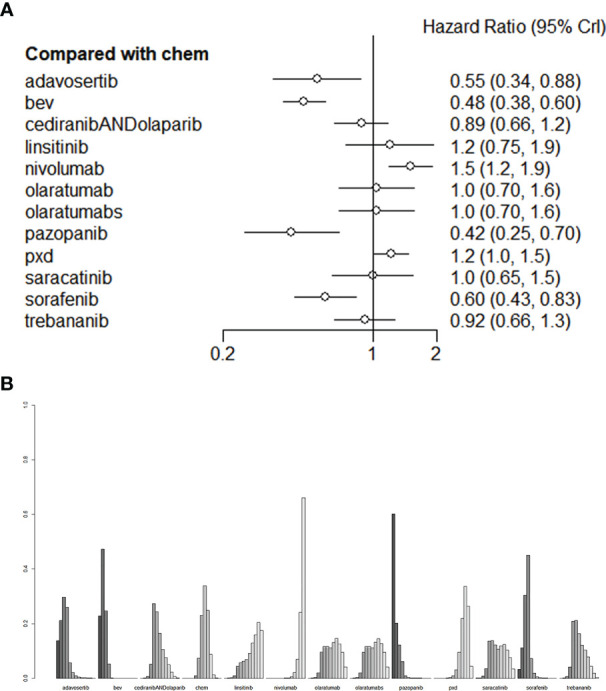
**(A)** The forest plot and **(B)** the ranking plot for PFS. The color of the lines in **Figure 5B** from dark to light represents the ranking from the best to the worst. A darker color represents a higher probability of becoming the best intervention, and a lighter color represents a lower probability. The cediranibANDcediranib stands for combination medication.

Compared with mono-chemotherapy, Bevacizumab + chemotherapy regimen (liposome doxorubicin or weekly paclitaxel or topotecan) and Adavosertib + Gemcitabine regimen would be the most effective for improving patients` PFS (**Figure 5A**), the HR of Bevacizumab and Adavosertib was 0.48 (95%CI:0.38-0.60) and 0.55 (95%CI:0.34-0.88), respectively, as shown in **Figure 5B**.

As for PFS, compared with chemotherapy, Adavosertib + Gemcitabine regimen and bevacizumab+ chemotherapy regiomen (liposome doxorubicin or weekly paclitaxel or topotecan) showed the best performance among all treatments (**Figure 5A**). Among these treatments, the HR of bevacizumab was 0.48 (95% CI: 0.38-0.60) and that of Adavosertib was 0.55 (95% CI: 0.34-0.88). The HR of Pazopanib and Sorafenib was 0.42(95%CI:0.25-0.70) and 0.60 (95%CI:0.43-0.83), with statistical significance. These two agents could be selected if Bevacizumab or Adavosertib is contraindicated. Bevacizumab and Adavosertib would be optimal for improving the PFS. All the comparisons of PFS are shown in **Figure 5B**. We performed validation through frequency-based network meta-analysis, and the results showed that Adavosertib + Gemcitabine regimen (HR= 0.55, 95%CI: 0.34 -0.88) and Bevacizumab + Gemcitabine regimen (HR= 0.48, 95%CI: 0.38-0.60) could significantly improve the PFS. Detailed results of frequency-based network meta-analysis are provided in **Appendix 2**.

**Figure 6:**
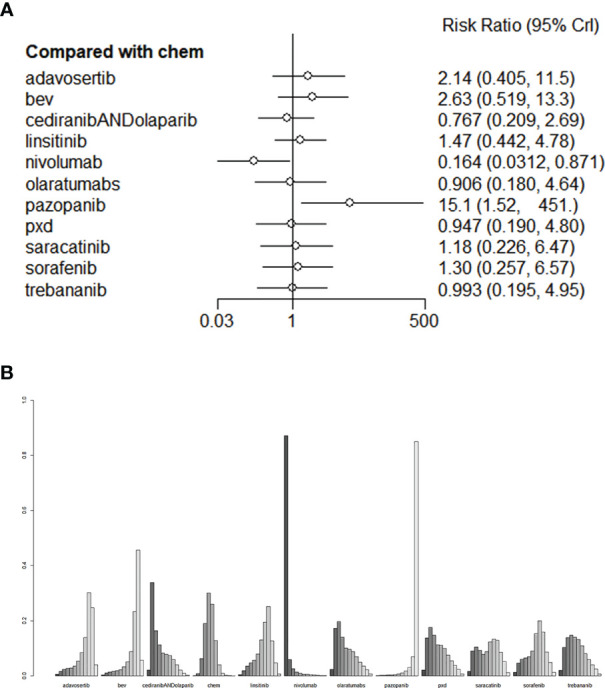
**(A)** The forest plot and **(B)** the ranking plot for AE. The color of the lines in **Figure 6B** from dark to light represents the ranking from the best to the worst. A darker color represents a higher probability of becoming the best intervention, and a lighter color represents a lower probability. The cediranibANDcediranib stands for combination medication.

As for AEs, Nivolumab had the lowest incidence of Grade 3-4 AEs (RR=0.0164, 95% CI: 0.0312-0.871). Pazopanib had the highest toxicity (RR = 15.1, 95% CI: 1.52-451). The league tables for AEs are presented in **Figure 6**. We also performed validation through frequency-based network meta-analysis, and the results showed that nivolumab (RR = 0.17, 95%CI:0.11-0.27) could significantly reduce the risk of AEs. Detailed results of frequency-based network meta-analysis are provided in **Appendix 2**.

## Discussion

In this study, we explored the best treatment options for recurrent platinum-resistant ovarian cancer through Bayesian network analysis including 11 RCTs involving 1687 patients. The findings were as follows:

1. Adavosertib + Gemcitabine/other chemotherapeutic agent regimens (liposome doxorubicin, weekly paclitaxel or topotecan) and Bevacizumab + Gemcitabine regimen were the most effective for recurrent PROC. These regimens were more beneficial to the patients comparing to mono-chemptherapy. Adavosertib + Gemcitabine regimen and Bevacizumab + Gemcitabine regimen presented evident merits in improving the patients` OS and PFS, so these two regimens would be the most beneficial to recurrent PROC patients.
2. For patients who are in a poor condition, too old, or unable to tolerate the above regimens, Pazopanib + weekly paclitaxel regimen or Sorafenib + topotecan could also improve their PFS.
3. Among all the assessed agents, Nivolumab has the mildest side effects, with the lowest incidence of grade 3-5 AEs. Nivolumab maintenance therapy could be considered for patients unable to tolerate chemotherapies due to their poor conditions or those in a stable condition.

Monotherapy often yields poor therapeutic effects for recurrent PROC, so that combination therapy has become increasingly crucial for the patients to improve their survival. We observed that Adavosertib, Bevacizumab, or immunotherapy combined with chemotherapeutic agents would lead to better survival than single targeted therapy, chemotherapy, or immunotherapy. This would be helpful to inhibit the cancer progression through targeting different pharmacological mechanisms.

Bevacizumab refers to a humanized monoclonal antibody targeting on vascular endothelial growth factor (VEGF), and is the most widely studied targeted agent for epithelial ovarian cancer (EOC). It exerts an inhibitive effect on the progression of recurrent PROC. One of the main characteristics of ovarian cancer was the massive angiogenesis that promotes tumor proliferation and metastasis, which could be inhibited by Bevacizumab through directly targeting on VEGF (30).

In this study, recurrent PROC patients receiving Bevacizumab + chemotherapy regimen had the best PFS, which might be attributed to the fact that Bevacizumab could increase the concentration of chemotherapeutic agents (31). AURELIA is the first phase-III trial that has compared the efficacy of Bevacizumab + chemotherapy with mono-chemotherapy for recurrent platinum-resistance (19). The combination of chemotherapeutic agents (weekly paclitaxel, liposome doxorubicin, and topotecan) with Bevacizumab led to a significantly increase in the objective omission rate (ORR) (12.6% vs 30.9%), and an improvement in the median PFS (6.7 vs 3.4), whereas the OS showed no significant difference. In this study, the combination of Bevacizumab with different chemotherapy regimens led to varied PRR and PFS (ORR: paclitaxel> topotecan> liposome doxorubicin; median PFS: paclitaxel> topotecan> liposome doxorubicin).

Other antiangiogenic agents that have been demonstrated effective for ovarian cancer in AURELIA include Nintedanib (32), Pazopanib (23, 33), Cediranib (34), Sorafenib (35), and angiogenin inhibitor Trebananib (20, 36). Pazopanib and Trebananib present remarkable effects, especially on PROC.

Pazopanib and Trebananib in combination with paclitaxel could improve the PFS (not the OS) (32, 34). In TRIAS study, patients receiving Sorafenib monotherapy had a very low ORR of 3% (37), which was increased (5%-7%) after combining Sorafenib with chemotherapy (38, 39). We observed that Topotecan + Sorafenib significantly improved that PFS of recurrent PROC patients. In addition, a study by Aya El Helali et al. (40), indicated that Pazopanib (P-score=0·79) and Sorafenib (P-score=0·76) administration combined with chemotherapy contributed to clinically significant improvement in the OS of recurrent PROC.

It was interesting that we observed Bevacizumab combined with chemotherapy also increased the PFS and OS of platinum-sensitive patients. A study by Yuanzhi Liu et al. (5) revealed that Bevacizumab in combination with platinum-based chemotherapy was more effective in improving the OS, PFS, and PRR of the patients (without BRCA mutation), compared with mono-chemotherapy. PARPi also showed its potential merits in the maintenance therapy for stable cancer. The ICON7 trial demonstrated that the use of bevacizumab for high-risk patients (>1 cm residual tumor) was associated with an improved OS (p=0.01, HR=0.78, 95%CI 0.63-0.97) (41). GOG-0218 trial demonstrated that for patients at FIGO stage-IV, combination of Bevacizumab with chemotherapy followed by Bevacizumab maintenance could be appropriate (HR=0.72, 95%CI 0.53-0.97) (42). Ray-Coquard, I. et al. (43) found that PARP inhibitors combined with antiangiogenic agents would be more beneficial for recurrent platinum-sensitive ovarian cancer, compared with monotherapy with antiangiogenic agents.

This Bayesian network meta-analysis indicated a significant difference in the PFS between recurrent PROC patients receiving Adavosertib + chemotherapy and those receiving Bevacizumab + chemotherapy (liposome doxorubicin, weekly paclitaxel, topotecan, or Gemcitabine), and the two regimen yielded a longest median survival, as well as the optimal therapeutic effects. Adavosertib + Gemcitabine could improve the OS, with statistical significance, while Bevacizumab + chemotherapy (topotecan) showed no statistical significance. This might be explained by different pharmacological mechanisms of these agents leading to different effects, which was validated in AURELIA trial. Different chemotherapy regimens yielded different therapeutic effects. We believe that the individualized selection of therapeutic agents for recurrent PROC patients proposed in this network meta-analysis would be of great significance.

Therefore, it could be seen from the above results that Adavosertib + chemotherapy was beneficial to both the PFS and OS in patients with recurrent PROC. Bevacizumab + chemotherapy could be applied not just for the primary and maintenance treatment of platinum-sensitive OC, but also for the treatment of recurrent PROC, to improve the PFS and partially improve the OS. Bevacizumab + chemotherapy could be selected if there is no need to consider drug resistance. Hence, Bevacizumab combined with chemotherapy has a survival benefit for platinum-sensitive patients with recurrent ovarian cancer.

Advantages and limitations: The advantages of this study were as follows: 1 This study discussed the best treatment options for recurrent platinum-resistant ovarian cancer and provided clinical significance for clinical practice. 2 The treatment plans in this study included the mainstream methods in current clinical practice. Limitations: 1 Although a comprehensive retrieval was conducted, the number of the included studies was still small. It was probably because recruiting participants with recurrent ovarian cancer remained to be a great challenge. 2 The number of studies on some mainstream treatment plans included in this analysis was small, and there might be events with small sample size. 3 There were more European and American subjects in the currently covered population, which might cause bias.

## Conclusion

In conclusion, Adavosertib, Bevacizumab, or antiangiogenic agents combined with chemotherapy (chemotherapy regimens are selected based on the patient’s previous medical history) might provide more benefits to patients with recurrent platinum-resistant ovarian cancer. Pazopanib + weekly paclitaxel regimen and Sorafenib + topotecan regimen could be considered if the above regimens are contraindicated, while the specific regimen should be made according to the individual conditions of the patients. Therefore, both efficacy and the incidence of adverse reactions should be considered, and the treatment plan should be made according to individual condition of patients. Current evidence needs to be interpreted. Different populations and regions of the patients should be cautiously taken into account. Prospective multicenter RCTs are thus needed to validate our recommendations.

## Data availability statement

The original contributions presented in the study are included in the article/**Supplementary Material**. Further inquiries can be directed to the corresponding author.

## Author contributions

JL, GZ and HY contributed to conception and design of the study and organized the database. WW, CY and SL performed the statistical analysis. JL and GZ wrote the first draft of the manuscript. WW, CY, HY and SL wrote sections of the manuscript. All authors contributed to manuscript revision, read, and approved the submitted version.
