# Supplementary material for: Treatment options for recurrent platinum-resistant ovarian cancer: A systematic review and Bayesian network meta-analysis based on RCTs
Source: Front Oncol. 2023 Apr 11;13:1114484. doi: 10.3389/fonc.2023.1114484 (PMC10126232; doi:10.3389/fonc.2023.1114484)
Supplement: Supplementary file 1 [file DataSheet_1.docx]

**APPENDIX 2 results of frequency-based network meta-analysis**

**1 Overall Survival**

Frequency-based network meta-analysis showed that: compared with conventional chemotherapy, Adavosertib (HR=0.56, 95%CI: 0.35-0.90), Guadecitabine (HR=0.72, 95%CI: 0.60-0.86), and Sorafenib (HR=0.65, 95%CI: 0.45-0.93) could significantly improve the OS, as shown in **Figure 1** and **Table 1**.


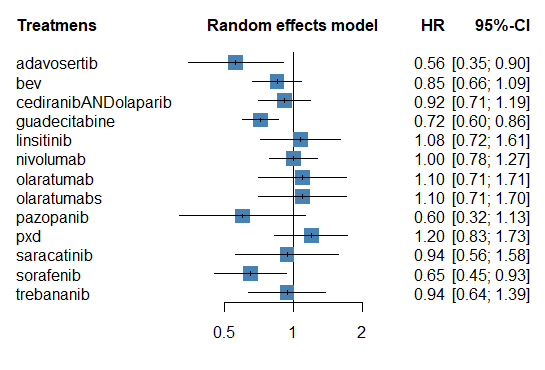


**Figure 1** Forest plot of frequency-based network meta-analysis for overall survival

**2 Progression-free Survival**

Frequency-based network meta-analysis showed that: compared with conventional chemotherapy, Adavosertib (HR=0.55, 95%CI: 0.34-0.88), Bevacizumab (HR=0.48, 95%CI:0.38-0.60), Pazopanib (HR=0.42, 95%CI:0.25-0.70), and Sorafenib (HR=0.60, 95%CI:0.43-0.83) could significantly improve the progression-free survival, as shown in **Figure 2** and **Table 2**.


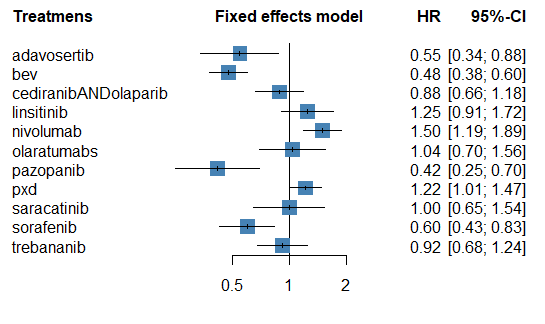


**Figure 2** Forest plot of frequency-based network meta-analysis for progression-free survival

**3 Adverse Events**

Frequency-based network meta-analysis showed that: compared with conventional chemotherapy, Nivolumab group had a lower incidence of adverse events (RR=0.17, 95%CI:0.11-0.27). However, Patients receiving Adavosertib (RR=2.06, 95%CI: 1.18-3.57), Bevacizumab (RR=2.62, 95%CI: 2.04-3.36), or Linsitinib (RR=1.48 95%CI: 1.00-2.19) have an increased risk of adverse events, as shown in **Figure 3** and Table 3.


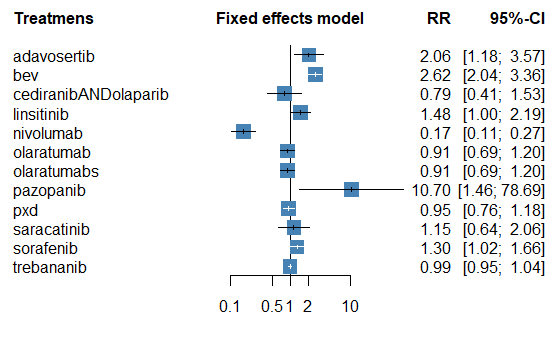


**Figure 3** Forest plot of frequency-based network meta-analysis for AEs

**Table 1** League table of frequency-based network meta-analysis for OS

| adavosertib | . | . |  | . | . | . | . | . | . | . | . | . | . |
| --- | --- | --- | --- | --- | --- | --- | --- | --- | --- | --- | --- | --- | --- |
| 0.66(0.38~1.13) | bev | . |  | . | . | . | . | . | . | . | . | . | . |
| 0.61(0.35~1.05) | 0.92(0.65~1.32) | cediranibANDolaparib |  | . | . | . | . | . | . | . | . | . | . |
| 0.56(0.35~0.90) | 0.85(0.66~1.09) | 0.92(0.71~1.19) | chem |  |  |  |  |  |  |  |  |  |  |
| 0.78(0.47~1.30) | 1.18(0.87~1.60) | 1.28(0.93~1.75) | 1.39(1.16~1.66) | guadecitabine | . | . | . | . | . | . | . | . | . |
| 0.52(0.28~0.97) | 0.79(0.49~1.27) | 0.85(0.53~1.38) | 0.93(0.62~1.39) | 0.67(0.43~1.04) | linsitinib | . | . | . | . | . | . | . | . |
| 0.56(0.33~0.96) | 0.85(0.60~1.20) | 0.92(0.64~1.31) | 1.00(0.78~1.27) | 0.72(0.53~0.97) | 1.08(0.67~1.72) | nivolumab | . | . | . | . | . | . | . |
| 0.51(0.27~0.98) | 0.77(0.47~1.28) | 0.84(0.50~1.40) | 0.91(0.59~1.42) | 0.66(0.41~1.06) | 0.98(0.54~1.78) | 0.91(0.55~1.51) | olaratumab | . | . | . | . | . | . |
| 0.51(0.27~0.98) | 0.77(0.47~1.28) | 0.84(0.50~1.40) | 0.91(0.59~1.41) | 0.66(0.41~1.05) | 0.98(0.54~1.78) | 0.91(0.55~1.50) | 1.00(0.54~1.87) | olaratumabs | . | . | . | . | . |
| 0.93(0.42~2.06) | 1.42(0.72~2.79) | 1.53(0.77~3.03) | 1.67(0.89~3.13) | 1.20(0.62~2.31) | 1.79(0.85~3.80) | 1.67(0.85~3.28) | 1.83(0.85~3.95) | 1.83(0.85~3.95) | pazopanib | . | . | . | . |
| 0.47(0.26~0.85) | 0.71(0.46~1.10) | 0.77(0.49~1.20) | 0.83(0.58~1.20) | 0.60(0.40~0.90) | 0.90(0.52~1.55) | 0.83(0.54~1.29) | 0.91(0.52~1.63) | 0.91(0.52~1.62) | 0.50(0.24~1.04) | pxd | . | . | . |
| 0.60(0.29~1.21) | 0.90(0.51~1.61) | 0.98(0.55~1.75) | 1.06(0.63~1.79) | 0.77(0.44~1.33) | 1.15(0.59~2.21) | 1.06(0.60~1.89) | 1.17(0.59~2.31) | 1.17(0.59~2.31) | 0.64(0.28~1.44) | 1.28(0.68~2.41) | saracatinib | . | . |
| 0.86(0.47~1.57) | 1.31(0.84~2.03) | 1.41(0.90~2.21) | 1.54(1.07~2.21) | 1.11(0.74~1.66) | 1.66(0.96~2.85) | 1.54(0.99~2.38) | 1.69(0.95~2.99) | 1.69(0.96~2.99) | 0.92(0.45~1.91) | 1.85(1.10~3.09) | 1.45(0.77~2.72) | sorafenib | . |
| 0.60(0.32~1.10) | 0.90(0.57~1.43) | 0.98(0.61~1.56) | 1.06(0.72~1.57) | 0.77(0.50~1.17) | 1.15(0.65~2.01) | 1.06(0.67~1.68) | 1.17(0.65~2.10) | 1.17(0.65~2.10) | 0.64(0.30~1.34) | 1.28(0.75~2.18) | 1.00(0.52~1.91) | 0.69(0.41~1.18) | trebananib |

**Table 1** League table of frequency-based network meta-analysis for PFS

| adavosertib | . | . |  | . | . | . | . | . | . | . | . |
| --- | --- | --- | --- | --- | --- | --- | --- | --- | --- | --- | --- |
| 1.15(0.68~1.94) | bev | . |  | . | . | . | . | . | . | . | . |
| 0.62(0.36~1.08) | 0.54(0.37~0.79) | cediranibANDolaparib |  | . | . | . | . | . | . | . | . |
| 0.55(0.34~0.88) | 0.48(0.38~0.60) | 0.88(0.66~1.18) | chem |  |  |  |  |  |  |  |  |
| 0.44(0.25~0.77) | 0.38(0.26~0.56) | 0.71(0.46~1.08) | 0.80(0.58~1.09) | linsitinib | . | . | . | . | . | . | . |
| 0.37(0.22~0.62) | 0.32(0.23~0.44) | 0.59(0.41~0.85) | 0.67(0.53~0.84) | 0.84(0.57~1.23) | nivolumab | . | . | . | . | . | . |
| 0.53(0.28~0.98) | 0.46(0.29~0.73) | 0.85(0.52~1.39) | 0.96(0.64~1.43) | 1.20(0.72~2.00) | 1.44(0.91~2.28) | olaratumabs | . | . | . | . | . |
| 1.31(0.65~2.62) | 1.14(0.66~1.99) | 2.11(1.17~3.78) | 2.38(1.43~3.96) | 2.98(1.64~5.42) | 3.57(2.05~6.23) | 2.48(1.30~4.74) | pazopanib | . | . | . | . |
| 0.45(0.27~0.75) | 0.39(0.29~0.53) | 0.73(0.51~1.03) | 0.82(0.68~0.99) | 1.03(0.71~1.48) | 1.23(0.91~1.65) | 0.85(0.55~1.33) | 0.34(0.20~0.59) | pxd | . | . | . |
| 0.55(0.29~1.04) | 0.48(0.29~0.78) | 0.88(0.53~1.49) | 1.00(0.65~1.54) | 1.25(0.73~2.14) | 1.50(0.92~2.45) | 1.04(0.58~1.88) | 0.42(0.22~0.82) | 1.22(0.76~1.95) | saracatinib | . | . |
| 0.92(0.52~1.63) | 0.80(0.54~1.19) | 1.47(0.95~2.29) | 1.67(1.20~2.32) | 2.09(1.33~3.29) | 2.50(1.67~3.73) | 1.74(1.03~2.92) | 0.70(0.38~1.28) | 2.03(1.39~2.97) | 1.67(0.97~2.87) | sorafenib | . |
| 0.60(0.34~1.05) | 0.52(0.36~0.76) | 0.96(0.63~1.46) | 1.09(0.80~1.47) | 1.36(0.88~2.11) | 1.63(1.12~2.38) | 1.13(0.69~1.87) | 0.46(0.25~0.82) | 1.33(0.93~1.89) | 1.09(0.64~1.84) | 0.65(0.42~1.02) | trebananib |

**Table 1** League table of frequency-based network meta-analysis for PFS

| adavosertib | . | . |  | . | . | . | . | . | . | . | . | . |
| --- | --- | --- | --- | --- | --- | --- | --- | --- | --- | --- | --- | --- |
| 0.79(0.43~1.44) | bev | . |  | . | . | . | . | . | . | . | . | . |
| 2.59(1.10~6.12) | 3.30(1.63~6.67) | cediranibANDolaparib |  | . | . | . | . | . | . | . | . | . |
| 2.06(1.18~3.57) | 2.62(2.04~3.36) | 0.79(0.41~1.53) | chem |  |  |  |  |  |  |  |  |  |
| 1.39(0.70~2.74) | 1.77(1.11~2.82) | 0.54(0.25~1.15) | 0.68(0.46~1.00) | linsitinib | . | . | . | . | . | . | . | . |
| 12.30(5.98~25.30) | 15.66(9.26~26.49) | 4.75(2.12~10.62) | 5.98(3.76~9.51) | 8.85(4.82~16.26) | nivolumab | . | . | . | . | . | . | . |
| 2.26(1.22~4.19) | 2.88(1.99~4.16) | 0.87(0.43~1.78) | 1.10(0.84~1.44) | 1.63(1.01~2.63) | 0.18(0.11~0.31) | olaratumab | . | . | . | . | . | . |
| 2.26(1.22~4.19) | 2.88(1.99~4.16) | 0.87(0.43~1.78) | 1.10(0.84~1.44) | 1.63(1.01~2.63) | 0.18(0.11~0.31) | 1.00(0.68~1.47) | olaratumabs | . | . | . | . | . |
| 0.19(0.02~1.52) | 0.24(0.03~1.83) | 0.07(0.01~0.61) | 0.09(0.01~0.69) | 0.14(0.02~1.06) | 0.02(0.00~0.12) | 0.09(0.01~0.64) | 0.09(0.01~0.64) | pazopanib | . | . | . | . |
| 2.17(1.20~3.93) | 2.76(1.98~3.85) | 0.84(0.42~1.68) | 1.05(0.85~1.32) | 1.56(0.99~2.45) | 0.18(0.11~0.29) | 0.96(0.68~1.36) | 0.96(0.68~1.36) | 11.29(1.52~84.04) | pxd | . | . | . |
| 1.78(0.80~3.98) | 2.27(1.21~4.27) | 0.69(0.29~1.66) | 0.87(0.49~1.55) | 1.28(0.64~2.59) | 0.14(0.07~0.30) | 0.79(0.42~1.50) | 0.79(0.42~1.50) | 9.28(1.16~74.16) | 0.82(0.44~1.53) | saracatinib | . | . |
| 1.58(0.86~2.89) | 2.01(1.42~2.85) | 0.61(0.30~1.23) | 0.77(0.60~0.98) | 1.14(0.72~1.81) | 0.13(0.08~0.22) | 0.70(0.48~1.01) | 0.70(0.48~1.01) | 8.23(1.10~61.42) | 0.73(0.52~1.01) | 0.89(0.47~1.66) | sorafenib | . |
| 2.07(1.19~3.61) | 2.64(2.05~3.40) | 0.80(0.41~1.55) | 1.01(0.96~1.06) | 1.49(1.00~2.22) | 0.17(0.11~0.27) | 0.92(0.69~1.21) | 0.92(0.69~1.21) | 10.79(1.47~79.36) | 0.96(0.76~1.20) | 1.16(0.65~2.08) | 1.31(1.02~1.68) | trebananib |
