# Supplementary material for: Treatment options for recurrent platinum-resistant ovarian cancer: A systematic review and Bayesian network meta-analysis based on RCTs
Source: Front Oncol. 2023 Apr 11;13:1114484. doi: 10.3389/fonc.2023.1114484 (PMC10126232; doi:10.3389/fonc.2023.1114484)
Supplement: Supplementary file 2 [file Table_1.docx]

**Appendix 1 |** Literature search strategy.

**1.PubMed**

| Search number | Query | Results |
| --- | --- | --- |
| #1 | Ovarian Neoplasms | 11 |
| #2 | "Ovarian Neoplasms"[Mesh] | 353 |
| #3 | ((((((((((((((((Ovarian Neoplasms[Title/Abstract]) OR (Ovarian Neoplasm[Title/Abstract])) OR (Ovary Neoplasms[Title/Abstract])) OR (Neoplasm, Ovary[Title/Abstract])) OR (Neoplasms, Ovary[Title/Abstract])) OR (Ovary Neoplasm[Title/Abstract])) OR (Neoplasms, Ovarian[Title/Abstract])) OR (Ovary Cancer[Title/Abstract])) OR (Cancer, Ovary[Title/Abstract])) OR (Cancers, Ovary[Title/Abstract])) OR (Ovary Cancers[Title/Abstract])) OR (Ovarian Cancer[Title/Abstract])) OR (Cancer, Ovarian[Title/Abstract])) OR (Cancers, Ovarian[Title/Abstract])) OR (Ovarian Cancers[Title/Abstract])) OR (Cancer of Ovary[Title/Abstract])) OR (Cancer of the Ovary[Title/Abstract]) | 77,800 |
| #4 | ("Ovarian Neoplasms"[Mesh]) OR (((((((((((((((((Ovarian Neoplasms[Title/Abstract]) OR (Ovarian Neoplasm[Title/Abstract])) OR (Ovary Neoplasms[Title/Abstract])) OR (Neoplasm, Ovary[Title/Abstract])) OR (Neoplasms, Ovary[Title/Abstract])) OR (Ovary Neoplasm[Title/Abstract])) OR (Neoplasms, Ovarian[Title/Abstract])) OR (Ovary Cancer[Title/Abstract])) OR (Cancer, Ovary[Title/Abstract])) OR (Cancers, Ovary[Title/Abstract])) OR (Ovary Cancers[Title/Abstract])) OR (Ovarian Cancer[Title/Abstract])) OR (Cancer, Ovarian[Title/Abstract])) OR (Cancers, Ovarian[Title/Abstract])) OR (Ovarian Cancers[Title/Abstract])) OR (Cancer of Ovary[Title/Abstract])) OR (Cancer of the Ovary[Title/Abstract])) | 5,512 |
| #5 | "Recurrence"[Mesh] | 0 |
| #6 | (((((Recurrence[Title/Abstract]) OR (Recurrences[Title/Abstract])) OR (Recrudescence[Title/Abstract])) OR (Recrudescences[Title/Abstract])) OR (Relapse[Title/Abstract])) OR (Relapses[Title/Abstract]) | 497,986 |
| #7 | ("Recurrence"[Mesh]) AND ((((((Recurrence[Title/Abstract]) OR (Recurrences[Title/Abstract])) OR (Recrudescence[Title/Abstract])) OR (Recrudescences[Title/Abstract])) OR (Relapse[Title/Abstract])) OR (Relapses[Title/Abstract])) | 195,655 |
| #8 | ("Recurrence"[Mesh]) AND ((((((Recurrence[Title/Abstract]) OR (Recurrences[Title/Abstract])) OR (Recrudescence[Title/Abstract])) OR (Recrudescences[Title/Abstract])) OR (Relapse[Title/Abstract])) OR (Relapses[Title/Abstract])) | 111,063 |
| #9 | ("Recurrence"[Mesh]) AND ((((((Recurrence[Title/Abstract]) OR (Recurrences[Title/Abstract])) OR (Recrudescence[Title/Abstract])) OR (Recrudescences[Title/Abstract])) OR (Relapse[Title/Abstract])) OR (Relapses[Title/Abstract])) | 68,227 |
| #10 | (("Ovarian Neoplasms"[Mesh]) OR (((((((((((((((((Ovarian Neoplasms[Title/Abstract]) OR (Ovarian Neoplasm[Title/Abstract])) OR (Ovary Neoplasms[Title/Abstract])) OR (Neoplasm, Ovary[Title/Abstract])) OR (Neoplasms, Ovary[Title/Abstract])) OR (Ovary Neoplasm[Title/Abstract])) OR (Neoplasms, Ovarian[Title/Abstract])) OR (Ovary Cancer[Title/Abstract])) OR (Cancer, Ovary[Title/Abstract])) OR (Cancers, Ovary[Title/Abstract])) OR (Ovary Cancers[Title/Abstract])) OR (Ovarian Cancer[Title/Abstract])) OR (Cancer, Ovarian[Title/Abstract])) OR (Cancers, Ovarian[Title/Abstract])) OR (Ovarian Cancers[Title/Abstract])) OR (Cancer of Ovary[Title/Abstract])) OR (Cancer of the Ovary[Title/Abstract]))) AND (("Recurrence"[Mesh]) AND ((((((Recurrence[Title/Abstract]) OR (Recurrences[Title/Abstract])) OR (Recrudescence[Title/Abstract])) OR (Recrudescences[Title/Abstract])) OR (Relapse[Title/Abstract])) OR (Relapses[Title/Abstract]))) | 91,229 |
| #11 | (("Ovarian Neoplasms"[Mesh]) OR (((((((((((((((((Ovarian Neoplasms[Title/Abstract]) OR (Ovarian Neoplasm[Title/Abstract])) OR (Ovary Neoplasms[Title/Abstract])) OR (Neoplasm, Ovary[Title/Abstract])) OR (Neoplasms, Ovary[Title/Abstract])) OR (Ovary Neoplasm[Title/Abstract])) OR (Neoplasms, Ovarian[Title/Abstract])) OR (Ovary Cancer[Title/Abstract])) OR (Cancer, Ovary[Title/Abstract])) OR (Cancers, Ovary[Title/Abstract])) OR (Ovary Cancers[Title/Abstract])) OR (Ovarian Cancer[Title/Abstract])) OR (Cancer, Ovarian[Title/Abstract])) OR (Cancers, Ovarian[Title/Abstract])) OR (Ovarian Cancers[Title/Abstract])) OR (Cancer of Ovary[Title/Abstract])) OR (Cancer of the Ovary[Title/Abstract]))) AND (("Recurrence"[Mesh]) AND ((((((Recurrence[Title/Abstract]) OR (Recurrences[Title/Abstract])) OR (Recrudescence[Title/Abstract])) OR (Recrudescences[Title/Abstract])) OR (Relapse[Title/Abstract])) OR (Relapses[Title/Abstract]))) | 1,759 |

**2.Cochrane**

| Search number | Query |
| --- | --- |
| #1 | MeSH descriptor: [Ovarian Neoplasms] explode all trees 2932 |
| #2 | (Ovarian Neoplasms):ti,ab,kw OR (Ovarian Neoplasm):ti,ab,kw OR (Ovary Neoplasms):ti,ab,kw OR (Neoplasm, Ovary):ti,ab,kw OR (Neoplasms, Ovary):ti,ab,kw. 4356 |
| #3 | (Ovary Neoplasm):ti,ab,kw OR (Neoplasms, Ovarian):ti,ab,kw OR (Ovary Cancer):ti,ab,kw OR (Cancer, Ovary):ti,ab,kw OR (Cancers, Ovary):ti,ab,kw. 6538 |
| #4 | (Ovary Cancers):ti,ab,kw OR (Ovarian Cancer):ti,ab,kw OR (Cancers, Ovarian):ti,ab,kw OR (Ovarian Cancer):ti,ab,kw OR (Ovarian Cancers):ti,ab,kw 8242 |
| #5 | (Cancer of Ovary):ti,ab,kw OR (Cancer of the Ovary):ti,ab,kw OR (ovary tumor):ti,ab,kw 3805 |
| #6 | #1 or #2 or #3 or #4 or #5 9340 |
| #7 | MeSH descriptor: [Ovarian Neoplasms] explode all trees 2932 |
| #8 | MeSH descriptor: [Recurrence] explode all trees 14408 |
| #9 | (Recurrence):ti,ab,kw OR (Recurrences):ti,ab,kw OR (Recrudescence):ti,ab,kw OR (Recrudescences):ti,ab,kw OR (Relapse):ti,ab,kw 81631 |
| #10 | (Relapses):ti,ab,kw 5810 |
| #11 | #8 or #9 or #10 183238 |
| #12 | #6 and #11 1618 |

**3.Embase**

| Search number | Query | Results |
| --- | --- | --- |
| #1 | 'ovary tumor'/exp | 4601 |
| #2 | 'ovary tumor':ab,ti OR 'ovarian neoplasms':ab,ti OR 'ovarian neoplasm':ab,ti OR 'ovary neoplasms':ab,ti OR 'neoplasm, ovary':ab,ti | 14147 |
| #3 | #1 OR #2 | 774866 |
| #4 | 'ovary tumor':ab,ti OR 'ovarian neoplasms':ab,ti OR 'ovarian neoplasm':ab,ti OR 'ovary neoplasms':ab,ti OR 'neoplasm, ovary':ab,ti OR 'neoplasms, ovary':ab,ti OR 'ovary neoplasm':ab,ti OR 'neoplasms, ovarian':ab,ti OR 'ovary cancer':ab,ti OR 'cancer, ovary':ab,ti OR 'cancers, ovary':ab,ti OR 'ovary cancers':ab,ti OR 'ovarian cancer':ab,ti OR 'cancer, ovarian':ab,ti OR 'cancers, ovarian':ab,ti OR 'ovarian cancers':ab,ti OR 'cancer of ovary':ab,ti OR 'cancer of the ovary':ab,ti | 179656 |
| #5 | #1 OR #4 | 95866 |
| #6 | recurrence:ab,ti OR recurrences:ab,ti OR recrudescence:ab,ti OR recrudescences:ab,ti OR relapse:ab,ti OR relapses:ab,ti | 169107 |
| #7 | #5 AND #6 | 2805 |
| #8 | #7 AND ('controlled clinical trial'/de OR 'controlled study'/de OR 'phase 2 clinical trial'/de OR 'phase 3 clinical trial'/de OR 'phase 3 clinical trial topic'/de OR 'randomized controlled trial'/de OR 'randomized controlled trial topic'/de) | 168851 |
| #9 |  | Results |
| #10 |  | 4601 |

**4.Web of science**

| Search number | Query |
| --- | --- |
| 1 | Ovarian Neoplasms (Topic) or Ovarian Neoplasm (Topic) or Ovary Neoplasms (Topic) or Neoplasm, Ovary (Topic) or Neoplasms, Ovary (Topic) or Ovary Neoplasm (Topic) 10,349 |
| 2 | Recurrence (Topic) or Recurrences (Topic) or Recrudescence (Topic) or Recrudescences (Topic) or Relapse (Topic) or Relapses (Topic) 603,005 |
| 3 | Recurrence (Topic) or Recurrences (Topic) or Recrudescence (Topic) or Recrudescences (Topic) or Relapse (Topic) or Relapses (Topic) 603,005 |
| 4 | Neoplasms, Ovarian (Topic) or Ovary Cancer (Topic) or Cancer, Ovary (Topic) or Cancers, Ovary (Topic) or Ovary Cancers (Topic) or Cancer, Ovarian (Topic) or Cancers, Ovarian (Topic) 26,254 |
| 5 | Recurrence (Topic) or Recurrences (Topic) or Recrudescence (Topic) or Recrudescences (Topic) or Relapse (Topic) or Relapses (Topic)603,005 |
| 6 | Ovarian Cancer (Topic) or Ovarian Cancers (Topic) or Cancer of Ovary (Topic) or Cancer of the Ovary (Topic) or ovary tumor (Topic)139.308 |
| 7 | Recurrence (Topic) or Recurrences (Topic) or Recrudescence (Topic) or Recrudescences (Topic) or Relapse (Topic) or Relapses (Topic)603,005 |
| 8 | Recurrence (Topic) or Recurrences (Topic) or Recrudescence (Topic) or Recrudescences (Topic) or Relapse (Topic) or Relapses (Topic)603,005 |
| 9 | #1 AND #2 1,211 |
| 10 | #9 AND #6 AND #4 1804 |
| 11 | #9 AND #6 AND #4 729 |
